# Elementary School-Based Influenza Vaccination: Evaluating Impact on Respiratory Illness Absenteeism and Laboratory-Confirmed Influenza

**DOI:** 10.1371/journal.pone.0072243

**Published:** 2013-08-26

**Authors:** Sonia A. Kjos, Stephanie A. Irving, Jennifer K. Meece, Edward A. Belongia

**Affiliations:** Marshfield Clinic Research Foundation, Marshfield, Wisconsin, United States of America; University of Hong Kong, Hong Kong

## Abstract

**Background:**

Studies of influenza vaccine effectiveness in schools have assessed all-cause absenteeism rather than laboratory-confirmed influenza. We conducted an observational pilot study to identify absences due to respiratory illness and laboratory-confirmed influenza in schools with and without school-based vaccination.

**Methods:**

A local public health agency initiated school-based influenza vaccination in two Wisconsin elementary schools during October 2010 (exposed schools); two nearby schools served as a comparison group (non-exposed schools). Absences due to fever or cough illness were monitored for 12 weeks. During the 4 weeks of peak influenza activity, parents of absent children with fever/cough illness were contacted and offered influenza testing.

**Results:**

Parental consent for sharing absenteeism data was obtained for 937 (57%) of 1,640 students. Fifty-two percent and 28%, respectively, of all students in exposed and non-exposed schools were vaccinated. Absences due to fever or cough illness were significantly lower in the exposed schools during seven of 12 surveillance weeks. Twenty-seven percent of students at exposed schools and 39% at unexposed schools had one or more days of absence due to fever/cough illness (p<0.0001). There was no significant difference in the proportion of students absent for other reasons (p = 0.23). During the 4 week period of influenza testing, respiratory samples were obtained for 68 (42%) of 163 episodes of absence due to fever or cough illness. Influenza was detected in 6 students; 3 attended exposed schools.

**Conclusions:**

Detection of laboratory-confirmed influenza in schools was challenging due to multiple consent requirements, difficulty obtaining samples from absent children, and a mild influenza season. School-based influenza vaccination was associated with reduced absenteeism due to fever or cough illness, but not absenteeism for other reasons. Although nonspecific, absence due to fever or cough illness may be a useful surrogate endpoint in school-based studies if identification of laboratory confirmed influenza is not feasible.

## Introduction

Children have high rates of influenza infection and increased duration of viral shedding, and serve as a major source of influenza transmission in communities [1], [2]. The prevention of influenza among children may be an effective method for reducing the overall burden of influenza [3], [4]. Routine annual influenza vaccination of all children aged 6 months through 18 years of age is recommended by the Advisory Committee on Immunization Practices (ACIP) [5], and school-based vaccination campaigns offer an opportunity to increase vaccine coverage in school-age children [6]. However, there is limited evidence that school-based vaccination leads to higher levels of protection against influenza in schools compared to current practices, which require parents to actively seek influenza vaccinations for their children [7], [8].

One of the earliest studies of vaccinating school children took place during the 1968–69 season in Tecumseh, Michigan [3]. Vaccination coverage of 86% was achieved in response to the campaign. In the outbreak of Hong Kong influenza (A/H3N2) that followed the campaign, rates of influenza-like illness were 3 times higher in a nearby control community compared to Tecumseh. School absenteeism rates were also substantially lower in Tecumseh. More recently, a county-wide vaccination program for elementary school students in Maryland demonstrated that absenteeism during the influenza season was reduced in the intervention county compared to two control counties [7]. However, reasons for absenteeism were not assessed, and absenteeism could not be compared between vaccinated and unvaccinated children. Similarly, King et al. examined the effect of school-based vaccination campaigns using a controlled study design [8]. Households with children in intervention schools reported lower absenteeism for influenza-like illness compared to households with children in control schools, but the change in total absenteeism did not differ significantly between the groups. These studies demonstrated the potential benefit of influenza vaccination in schools, but they were not designed to estimate the direct and indirect effects of school-based vaccination for preventing laboratory-confirmed influenza.

Studies involving vaccination and follow-up of children in school settings can be resource intensive, but the potential public health benefit is great if school-based interventions can reduce influenza-related absenteeism and community-wide transmission. We conducted an observational pilot study to assess the feasibility of identifying absences due to laboratory-confirmed influenza in vaccinated and unvaccinated students, and to compare absenteeism due to fever or cough illness in schools with and without the school-based vaccination initiative.

## Methods

### Setting

This study was conducted in Marshfield, Wisconsin, a city with approximately 20,000 residents in a predominantly rural area of the state. This was an observational study of a public health activity (school-based vaccination), and there was no research intervention or group randomization. The Wood County Health Department (WCHD) selected two elementary schools and the middle school (grades 7 and 8) in the district for school vaccine clinics out of a total of six public schools (four elementary, 1 middle school, 1 high school) located within the city of Marshfield. Vaccine clinics were not offered at other schools because WCHD did not have sufficient resources to provide vaccinations at all schools in the district. The observational study was restricted to the elementary schools.

For the school-based vaccination program, WCHD distributed an information letter, Vaccine Information Statement, and consent form to parents of all children at both schools. Consenting parents had the option to select trivalent inactivated vaccine (TIV), live attenuated influenza vaccine (LAIV), or no preference for vaccine type. Screening questions identified children with a contraindication to one or both vaccines, and these children were not eligible for vaccination. Signed consent forms were required by WCHD at least 3 days before the scheduled school vaccination clinic; no children were vaccinated without a signed consent form. Parents were not required to be present at the time the child was vaccinated. Each school was visited on two separate dates to provide two doses when necessary and ensure that as many students as possible were vaccinated. Vaccination clinics were held in schools on October 5–6 and November 9–10, 2010. Parents of children in every elementary school had access to influenza vaccination through primary health care providers at the Marshfield Clinic and vaccination clinics offered in other venues.

Elementary schools A and B, which held the school-based vaccination clinics, were designated as the exposed group. The two remaining elementary schools (C and D) located within the city of Marshfield were designated as the non-exposed group. Total student enrollment for the exposed and non-exposed schools was 982 and 658, respectively.

### Influenza Vaccination Status

All children attending Marshfield public schools have comprehensive vaccination records, including influenza vaccinations, in a web-based vaccination registry (RECIN) that is used by all private and public vaccination providers in the area (www.recin.org) [9]. A validation study of the registry during the 2006–07 and 2007–08 influenza seasons demonstrated that the registry captured 95% of all influenza vaccinations in the local population [10]. A subsequent validation study during the 2010–11 influenza season demonstrated that the registry captured 99% of all influenza vaccinations given to children age 5–17 years old who lived in the community (unpublished data). Children were classified as vaccinated beginning 14 days after influenza vaccine receipt. Children less than 9 years old were classified as fully vaccinated only if they had received two or more lifetime doses of influenza vaccine. Children less than 9 years of age were classified as partially vaccinated if they had received only one of two recommended doses.

Individual-level influenza vaccination information as of January 3, 2011 was obtained for all children whose parent(s) signed a Family Educational Rights and Privacy Act (FERPA) consent form. This consent was required for the school district to provide individual student information to the investigators, including dates of absence from school and vaccination status. Summary data on influenza vaccination coverage was available by grade for children attending each of the four study schools.

### Enrollment and Absenteeism Surveillance

Surveillance for absenteeism due to respiratory illness and other causes was initiated at each of the four schools on January 3, 2011, prior to widespread circulation of influenza in central Wisconsin. Daily surveillance of absenteeism continued for 12 weeks and included the period of peak influenza activity. Prior to the surveillance period, letters were sent home with students to solicit parental FERPA consent allowing release of individual absenteeism data to the researchers. A second letter, which included a self-addressed, stamped envelope, was distributed four weeks after the initial letter in an attempt to maximize the response rate. All families received two telephone reminders regarding the absenteeism consent form. Additional promotion for the study included articles released in two consecutive quarterly school newsletters (fall and winter 2010) and an informational booth display at each study school during the annual open houses held prior to start of classes each fall.

The four participating schools recorded daily all-cause absenteeism and absenteeism specifically due to fever or cough for the 12 week surveillance period. Individual absences (for FERPA-consented students) and total daily absenteeism counts (for all students) were reported by schools each day using a custom Microsoft Access application. Training was provided to school administrative staff who were responsible for tracking daily absences. In some cases, parents left voice messages to indicate student absences, and a school staff member called the parent to determine if the absence was due to fever or cough illness or other reasons. Only full-day absences were recorded by the schools. Students who were sent home early due to illness were not counted as absent on that day.

### Recruitment for Influenza Testing

Due to resource constraints, influenza testing of students was restricted to a 4-week period that included the peak of the influenza season in central Wisconsin. Influenza testing of students was initiated on February 7, 2011, based on increasing detection of influenza among patients tested in the outpatient setting for a separate study of vaccine effectiveness [11]. Recruitment for influenza testing was restricted to those students whose parents had signed a FERPA consent form, which allowed daily monitoring of school absences. The names of FERPA-consented students who were absent due to fever or cough illness were reported to study staff by the schools each day. Parents were then contacted by phone by a trained research coordinator to determine eligibility and request parental consent to collect a swab for influenza testing. Students were eligible to participate if their illness included fever or cough, duration from symptom onset to enrollment was ≤7 days, and they had not been enrolled in the study in the previous 14 days. Children were not eligible if they were taking an antiviral medication (i.e., oseltamivir). Multiple absent days due to fever or cough illness were considered a single episode if they occurred within a 14 day window.

Nasal and throat swabs were collected from children consenting to specimen collection. The nasal swab was collected from children by inserting a large Dacron- or rayon-tipped swab into one nostril to a depth of 2–3 cm, rotating gently against the septum for 3–5 seconds and then withdrawing. The throat swab was collected by gently wiping the posterior throat and tonsil area with a large Dacron or rayon tipped swab. Nose and throat swabs were combined in M4-RT viral transport media for influenza testing. All swabs were collected at participants’ homes or in the clinic by trained research coordinators.

This study was reviewed and approved by the Marshfield Clinic Institutional Review Board. Written informed consent was obtained from the parent or legal guardian of all participants prior to specimen collection. Verbal assent for collection was obtained from children who were at least 7 years old.

### Laboratory Methods

All enrollment samples were tested for influenza using real-time reverse transcription polymerase chain reaction (rRT-PCR). Centers for Disease Control and Prevention (CDC) primers, probes, and procedures were used for this assay. rRT-PCR was performed on nucleic acid extracts using the LightCycler® Real-Time PCR System (Roche Diagnostics, Basel, Switzerland). Total nucleic acid isolation and purification was performed using automated magnetic bead technology (MagNA Pure). The rRT-PCR assay is a TaqMan® based real-time detection of the matrix protein (M1) of influenza A and the non-structural protein 1 (NS1) of influenza B. Both have been shown to be highly conserved, representing effective targets for detection.

### Statistical Analysis

Influenza vaccine coverage was compared for the exposed and non-exposed schools. The weekly incidence proportion of absenteeism was calculated at the exposed and non-exposed schools for two types of absenteeism: (1) absences due to fever/cough illness; and (2) other-cause absenteeism. The incidence proportion was calculated as the number of students absent on one or more days in a given week (numerator) divided by the number of enrolled students (denominator). Chi-square tests were used to assess differences in vaccination, parental consent, and absenteeism at the exposed and non-exposed schools. Student enrollment for calculating absenteeism rates was based on enrollment counts as of September 1, 2011 (1,640 students), and FERPA-consented students were assumed to be continuously enrolled in school for the duration of the study. Student enrollment for calculating vaccination coverage was based on enrollment counts as of January 3, 2011 (1,659), when vaccination data were retrieved.

The primary objective included comparison of rRT-PCR-confirmed influenza attack rates among students attending the exposed vs. non-exposed schools. However, few cases of laboratory-confirmed influenza were detected during the four week testing period, and the primary analysis compared rates of absenteeism due to fever or cough illness instead. The intent of this study was to assess the feasibility of implementing a larger study rather than hypothesis testing, and power calculations were not performed. All statistical tests were performed in SAS 9.2 software (SAS Institute Inc., Cary, North Carolina).

## Results

There were 1,640 students enrolled at the four elementary schools as of September 1, 2010. Of these, 937 (57%) received parental (FERPA) consent which allowed the school district to record their individual absences for the study. The proportion of FERPA-consented students was similar at the exposed schools (n = 551, 56%) and the non-exposed schools (n = 386, 59%) (p = 0.31). The majority of signed consent forms (84%) were collected following the first distribution of the letter to parents. Vaccination coverage for all students enrolled in the study schools on January 3, 2011 is shown in Table 1. Fifty-two percent of all students at the exposed schools and 28% of those attending the non-exposed schools were fully vaccinated against influenza (p<0.001). At the exposed schools, the school-based vaccine clinics accounted for 65% of influenza vaccines received by students.

**Table 1:** Influenza vaccination status for students with and without FERPA consent to monitor absences.

Influenza Vaccination status	Exposed schools			Non-exposed schools			All Schools
	Consented*	Non-consented	Total	Consented*	Non-consented	Total	
	No. (%)	No. (%)	No. (%)	No. (%)	No. (%)	No. (%)	No. (%)
Fully vaccinated	350 (63.5)	171 (38.2)	521 (52.2)	124 (32.1)	58 (21.2)	182 (27.6)	703 (42.4)
Partially vaccinated	39 (7.1)	34 (7.6)	73 (7.3)	35 (9.1)	25 (9.1)	60 (9.1)	133 (8.0)
Not vaccinated	162 (29.4)	243 (54.2)	405 (40.5)	227 (58.8)	191 (69.7)	418 (63.3)	823 (49.6)
Total	551	448	999	386	274	660	1659

* students with parental FERPA consent.

Vaccination status and enrollment was determined as of January 3, 2011.

Absenteeism due to fever or cough illness was significantly lower in the exposed schools relative to the non-exposed schools during seven of the 12 surveillance weeks. Absenteeism due to other causes was similar at exposed and non-exposed schools with the exception of two weeks where the non-exposed school rates were significantly higher than the exposed school rates. The proportion of absences due to unknown cause ranged from 0.2–1.9% per week at the exposed schools and 0.5–2.8% at the non-exposed schools, and accounted for 6.4% and 6.7% of all absences during the 12 week period at the exposed and non-exposed schools, respectively.

During the 12-week surveillance period, there was a significant difference in the proportion of FERPA-consented students who were absent for at least one day due to fever or cough illness at the exposed vs. non-exposed schools (27 and 39%, respectively; p<0.0001) (Table 2). There was no significant difference in the proportion of FERPA-consented students with absences due to other causes at the exposed vs. non-exposed schools (43% and 47%, respectively; p = 0.23) (Table 2). FERPA-consented students who were unvaccinated were not more likely to be absent due to fever or cough illness compared to students who were fully vaccinated at either the exposed (p = 0.23) or non-exposed schools (p = 0.41) (Table 3).

**Table 2:** Association between school type and absenteeism among FERPA-consented students.

	Absent due to fever or cough illness			Absent due to other causes		
	Exposed Schools	Non-exposed Schools	Odds Ratio (95% CI)	Exposed Schools	Non-exposed Schools	Odds Ratio (95% CI)
	No. (%)	No. (%)		No. (%)	No. (%)	
Absent ≥1 day	146 (26.5)	150 (38.9)	1.76 (1.33, 2.33) p<0.0001	235 (42.7)	180 (46.6)	1.18 (0.9, 1.53) p = 0.23
Never absent	405 (73.5)	236 (61.1)		316 (57.4)	206 (53.4)	

**Table 3:** Influenza vaccination status and absences due to fever or cough among FERPA-consented students at exposed and non-exposed schools.

	Exposed schools			Non-exposed schools		
	Absent due to fever or cough illness	Absent due to other causes or never absent	Odds Ratio(95% CI)	Absent due to fever or cough illness	Absent due to other causes or never absent	Odds Ratio(95% CI)
	No. (%)	No. (%)		No. (%)	No. (%)	
Fully Vaccinated	84 (64.1)	266 (69.8)	0.77 (0.5, 1.2) p = 0.23	52 (38.0)	72 (33.7)	1.2 (0.75, 1.9) p = 0.41
Not vaccinated	47 (35.8)	115 (30.2)		85 (62.0)	142 (66.4)	

Vaccination status was determined by an immunization registry; students who were partially vaccinated were excluded.

During the four week influenza testing period from February 7 to March 4, 2011, 163 episodes of absence due to fever or cough illness (among 155 students) were recorded for FERPA-consented students at the four schools. Eight students had two episodes of absence due to fever or cough illness. The proportion of FERPA-consented students with absence due to fever or cough illness during this four week period was 17% at both the exposed and non-exposed schools. Follow-up telephone interviews with parents/guardians identified 72 illnesses that were eligible for influenza testing. Of the remaining 91 episodes, 30 were ineligible, 38 refused to participate, and 23 were unreachable by telephone. The most common reasons for an ineligible episode included parent-reported absence of fever or cough illness (n = 16), language barrier (i.e., non-English speaking parent or guardian, n = 5), and duration of symptoms greater than seven days (n = 3). The explanation for the discrepancy between parent-reported and school-reported reason for absence was not determined for the 16 children whose parents denied the presence of fever or cough illness during a phone interview.

Consenting students were enrolled and swabbed for 68 of the 72 eligible episodes of absence due to fever/cough. Of the 68 episodes, six (9%) were rRT-PCR positive for influenza, including 3 (7%) of 46 attending exposed schools and 3 (14%) of 22 attending non-exposed schools. Of the rRT-PCR positive samples, three were A (H3N2) and three were type B. Overall, the proportion of FERPA-consented students with absence due to laboratory-confirmed influenza was 0.54% at the exposed schools and 0.77% at the non-exposed schools.

## Discussion

The results from this observational study demonstrated that school-based clinics sponsored by a local public health agency led to increased vaccine coverage among elementary school students. In the exposed schools, the proportion of students who received at least one dose of vaccine was nearly two fold higher than the proportion vaccinated at the non-exposed schools. Overall vaccine coverage exceeded 50% at these schools, and vaccines administered in the school clinics accounted for nearly two-thirds of all vaccinated children at these schools. Similar levels of vaccine coverage have been reported from school-based vaccination clinics in other studies. In an intervention study involving 11 elementary schools in four states, 47% of students in the intervention schools vs. 2% in the control schools received LAIV [8]. In a nonrandomized county-level study in Maryland, 44% of students at 21 intervention schools received LAIV [7]. In the current study, parents had the option to choose TIV or LAIV if there were no contraindications, and it has been suggested that offering a choice of vaccine may produce higher vaccination rates compared to offering either vaccine alone [6]. Differences in parent recruitment methods, consenting procedures, and community or school characteristics may also influence the success of school-based vaccination clinics.

We were able to track aggregate absenteeism (without identifiers) due to fever/cough illness for all students, including those without FERPA consent. Absenteeism due to fever or cough illness was significantly reduced in exposed schools vs. non-exposed schools. In contrast, absenteeism due to other causes (i.e., not fever or cough) was similar at the exposed and non-exposed schools. These findings suggest that absenteeism due to fever or cough illness during periods of influenza circulation may be a useful endpoint to monitor in larger school-based studies when influenza diagnostic testing is not feasible. However, absenteeism is a nonspecific outcome and more susceptible to bias and confounding compared to rRT-PCR confirmed influenza. Unfortunately, we were unable to compare absenteeism due to fever or cough illness in the schools during periods when influenza was not circulating, since the schools did not record the reason for absence prior to initiation of the study.

Identifying absences due to rRT-PCR confirmed influenza was challenging due to lack of FERPA consent for many students, difficulty contacting parents, and the occurrence of a relatively mild influenza season. We had anticipated a high level of parental FERPA consent to monitor absenteeism, which would allow us to contact parents by phone and offer influenza testing whenever a child was absent with an illness that included fever or cough. We actively recruited and informed parents through a variety of methods, including letters sent home with students, attendance at school open houses, and automated telephone reminders. Despite these efforts, over 40% of students did not have parental consent to monitor individual absenteeism, although the proportion consenting was similar at the exposed and non-exposed schools. In the absence of parental consent, a large proportion of students could not be identified or offered influenza testing if they were absent due to fever or cough. Comments from teachers suggested that some parents at the exposed schools were confused by receipt of multiple consent forms, including a vaccination consent from the local health department, and a FERPA consent form from Marshfield Clinic for monitoring absenteeism. Some parents reportedly believed that completion of one form gave permission for participation in both activities. However, this did not account for the low proportion consenting at the non-exposed schools where vaccine clinics were not held.

An additional limitation was that students with parental FERPA consent represented a biased sample of all students attending these schools. The consented students at both the exposed and non-exposed schools had higher influenza vaccine coverage compared to their non-consented counterparts, and absenteeism due to other causes (i.e., not fever or cough) was higher among non-consented students. Absenteeism due to fever or cough illness was similar among consented and non-consented students at the exposed schools, but not at the non-exposed schools. The reason for this difference is not apparent. The requirement for FERPA consent was directly related to the study goal of identifying cases of rRT-PCR confirmed influenza among students absent with fever or cough. Eliminating any contact with individual students and parents would have avoided the need for FERPA consent because personal identifiers would not have been needed. For example, a recent study of A (H1N1)pdm09 monovalent vaccine administration in Maine elementary schools used school absenteeism as the endpoint without utilizing individual-level data [12]. However, assessment of all-cause absenteeism can lead to substantial outcome misclassification and underestimation of vaccine effects relative to a specific endpoint such as rRT-PCR confirmed influenza.

Our experience with this pilot study suggests that larger school-based studies using rRT-PCR confirmed influenza as the primary endpoint may be difficult to implement and resource intensive. One study addressed this challenge during the 2009 pandemic by using a case-control design with influenza cases identified through clinical rRT-PCR testing and laboratory-based reporting [13]. Controls were sampled from classmates without influenza-like illness who were present in school. Such a design can be useful to estimate direct vaccine effects, but the indirect and combined effects of school-based vaccination cannot be assessed. Although the optimal study design remains uncertain, further evaluation of school-based influenza vaccination is needed to determine its impact at the individual, school and community level.
